# The Sale of Poisonous Drugs

**Published:** 1881-05

**Authors:** 


### Editorial.

### THE SALE OF POISONOUS DRUGS.

It is one of the functions of civil government to afford the fullest protection to life and property. Whatever may jeopardize the safety of the individual, or may impair the industries by which communities are supported, diminishes the wealth and prosperity, which it is the special duty of the State to foster and encourage. These plain axioms are called to mind as we read the announcement in the secular press of suicides from poisonous drugs, which of late have occurred with rather alarming frequency. Whatever may be the antecedent causes leading to this suicidal mania, which it is not our purpose to consider at this time, it may be well worth the attention of the profession and the public to investigate the means of self-destruction, usually employed, and especially the legal safeguards existing for the prevention of this wanton destruction of human life.

The suicide of a popular business man, in this city, by an over-dose of tincture of opium, taken with the full purpose of self-destruction, in order to escape financial embarrassment unwisely assumed, illustrates the facility with which the most virulent poisons can be obtained from our druggists, and the fatal consequences often following their sale.

The law regulating the sale of the poisons affords an inadequate barrier against the great dangers involved in this important question. It simply requires the druggist to keep a "Poison Register," in which the name and residence of the purchaser, and the special objects for which the poison is to be employed are recorded. In the case above referred to, one or more bottles, marked with the fatal drug were found in his apartment, and also the name of the druggist from whom it was obtained. We do not doubt that the vender observed all the legal requirements in question.

This instance is one only of many in which the weapon to be used in self-destruction, is put into hands that secretly defy the laws both of God and man; and the inquiry is naturally suggested, is not the law, as it now stands upon the statute book, so imperfect that it affords no adequate restraint to the sale of most dangerous agents, and therefore an uncertain protection to human life?

We recognize the difficulties with which the pharmacists have to encounter, and therefore withhold indiscriminate censure. The real object of the purchaser may be withheld and the credibility of his statements, at the time of sale, not easily determined. If doubt exists, would it not be well to take advantage of the suspicion and act accordingly. We give our pharmaceutical friends the credit of possessing a conscience in this and kindred matters, even though it may be swayed at times by the spirit of avarice, a not infrequent failing of human nature, of which their patrons often and with seeming justice complain.

The great interests of human life, the most sacred of any with which the State is called upon to legislate, demand further safeguards and more severe restrictions in the sale of fatal drugs. We think the medical profession should assume this responsibility, and if druggists were compelled by law to require a physician's prescription, when called upon by his customer for any agent which is destructive of human life, when taken in large doses, the responsibility would rest where it justly belongs; and if perchance the agent so desired should be employed for suicidal purposes, the physician would be held by the public to a strict accountability for the abuse of the trust, thus committed to him. This is the only solution of this question. It imposes a duty upon the profession, which by the very nature of our calling, and by the training to which we are subjected, it is expected we will be qualified to perform. It places the only effectual check to a dangerous traffic which in the light of events of daily occurrence, it is full time, should be promptly instituted.

